# Prioritising and ranking methodological uncertainties in the design, conduct, and analysis of trials aiming to improve health outcomes for those living with multiple long-term conditions: A protocol for a modified Delphi study

**DOI:** 10.1177/26335565261459136

**Published:** 2026-06-08

**Authors:** Ayat Ahmadi, Lisong Zhang, Natasha Bryant, Natalie Darko, Kamlesh Khunti, Sally J Singh, Sharon A Simpson, Susan M Smith, Rod S Taylor, Miles D Witham, Hannah Young, Laura J Gray

**Affiliations:** 1 Division of Public Health and Epidemiology, 573772 University of Leicester, Leicester, UK; 2 573772 NIHR Leicester Biomedical Research Centre, University of Leicester, Leicester, UK; 3 NIHR Applied Research Collaboration East Midlands, 573772 University of Leicester, Leicester, UK; 4 Leicester British Heart Foundation Centre of Research Excellence, 573772 University of Leicester, Leicester, UK; 5 Diabetes Research Centre, 573772 University of Leicester, Leicester, UK; 6 Department of Respiratory Sciences, 573772 University of Leicester, Leicester, UK; 7 Public Health, School of Health and Well Being, 47970 University of Glasgow, Glasgow, UK; 8 Discipline of Public Health and Primary Care, 8809 Trinity College, Dublin, Ireland; 9 Robertson Centre for Biostatistics, School of Health and Well Being, 150855 University of Glasgow, Glasgow, UK; 10 AGE Research Group, Translational and Clinical R esearch Institute, Faculty of Medical Sciences, 5994 Newcastle University, Newcastle upon Tyne, UK; 11 NIHR Newcastle Biomedical Research Centre, Newcastle upon Tyne Hospitals NHS Foundation Trust, 5994 Cumbria Northumberland Tyne and Wear NHS Foundation Trust and Faculty of Medical Sciences Newcastle University, Newcastle upon Tyne, UK

**Keywords:** multimorbidity, research design, clinical trial, consensus

## Abstract

**Introduction:**

Multiple long-term conditions (MLTCs), defined as the co-existence of two or more chronic health conditions, are increasingly prevalent across all age groups and disproportionately affect socioeconomically disadvantaged and ethnic minority populations. Trials targeting MLTCs face methodological challenges due to patient heterogeneity, variation in selection of conditions and limitations in design and analysis. These challenges may contribute to the lack of evidence to inform effective interventions for people with MLTCs. This study aims to systematically identify and prioritise key methodological uncertainties in the design, conduct and analysis of future trials aiming to improve health outcomes for people with MLTCs.

**Methods:**

We will conduct a four-round modified Delphi study involving key interest groups including methodologists, trialists, MLTC researchers, research funders, commissioners, regulators, people with MLTC lived experience and MLTC carers. The process will include two rounds of online questionnaires, one lived-experience meeting (in-person or virtual) and a final virtual consensus meeting. To promote inclusivity and diversity of input, participants may join at any stage. Responses from each round will be analysed and summarised to inform the next stage, with final priorities agreed through structured consensus voting.

**Expected Outcomes:**

This consensus study will produce a ranked list of methodological research uncertainties to guide future research and clinical trial design, conduct and analysis in MLTCs. Findings will be disseminated through a facilitated online dissemination workshop, academic channels and targeted public engagement activities to support inclusive and relevant MLTC research.

## Strengths and limitations of this study

• There is limited guidance on how to design, conduct and analyse trials that aim to improve outcomes for people living with Multiple Long-Term Conditions (MLTCs). This consensus study will identify the priority methodological issues in this area.
• The study integrates Patient and Public Involvement (PPI) at every stage, from study design to dissemination, ensuring that the voices of people with lived experience are central to the process and outcomes.
• For encouraging greater inclusivity and diversity of perspectives, especially from individuals who may face time or accessibility constraints, we will apply a modified Delphi method which allows participants to join in different rounds. Facilitated meetings with people with lived experience and carers will ensure the final priorities are relevant to those with MLTCs.
• Allowing different participants in each round may lead to variability in responses and make it harder to establish consensus across rounds.
• The broad inclusion criteria and reliance on self-selection may result in underrepresentation of some interest groups, particularly among people with lived MLTC experience or underserved communities.

## Introduction

Multiple long-term conditions (MLTCs), commonly referred to as multimorbidity, describes the co-existence of two or more long-term conditions (LTCs), which may include non-communicable diseases, chronic mental health conditions, or persistent infectious diseases.^1,2,3–5^ While traditionally associated with ageing, recent studies indicate that MLTCs are common in midlife as well as old age, with a younger age of onset and higher prevalence observed among ethnic minority groups and socioeconomically deprived populations.^6,7^ These individuals experience high treatment burden, poorer health-related quality of life (HRQoL), and increased premature mortality.^8–11^ The complexity of MLTCs, along with the limited number of clinical trials, often with mixed results, contribute to the lack of evidence on the effectiveness of interventions for the management of people with MLTCs. Therefore, there is a clear need for rigorous studies to evaluate the efficacy and effectiveness of such interventions.^1,12^

Clinical trials involving people with MLTCs differ from traditional trials focused on single conditions in several ways. A key distinction lies in the heterogeneity of the included participants, who can have a wide range of LTCs which combine in many ways, compared to single condition focussed trials which tend to have strict inclusion and exclusion criteria giving a more homogenous sample.^1^ More conditions usually also means more background interventions, including medications, lifestyle changes and condition monitoring, all of which may interact with any new intervention being tested. These factors, along with coexisting activity limitation, symptom burden and a higher chance of acute intercurrent illness, all present barriers to trial participation and may increase the risk of dropout from trials. Beyond concerns about heterogeneity and burden of conditions, methodological challenges also exist in selecting appropriate outcomes (choice of generic versus disease specific outcomes), accounting for potential interactions between coexisting conditions and intervention effectiveness, higher adverse event rates, and statistical analysis that appropriately accounts for population heterogeneity, drop out and noncompliance.^13,14^

We have recently completed a systematic review of MLTC trials which interrogated the methodologic approach. The protocol for the systematic review has been published.^15^ The review showed a notable increase in MLTC clinical trials since 2010. Most studies employed individually randomised, two-arm parallel designs with usual care as the comparator, predominately conducted in primary care or community settings. Outcomes varied, but frequently included medication use, quality of life, healthcare utilisation, and condition-specific improvements. While intention-to-treat analysis was widely used, sensitivity analyses and handling of missing data were less consistently addressed. The review suggests that the studies conducted to date use traditional designs, with no consideration of more innovative designs and approaches (link).

It may be that the methodologies used to date have contributed to the limited evidence of effectiveness. Currently there is a lack of methodological guidance to inform the conduct, design and analysis of MLTC studies.^16,17^ To inform good research practice for future trials in this area in a systematic way, we will conduct a modified Delphi study to identify and prioritise methodological uncertainties in the design, conduct and analysis of trials aiming to improve outcomes in people with MLTCs, in order to guide future methodological research. The primary output of this study is expected to be a comprehensive list of methodological research uncertainties that will inform future methodological research. This study will focus on methodological challenges rather than specific MLTC research questions, which has been the focus of a previously completed Delphi study.^18^ The Delphi method was chosen as an efficient way to enable input from a range of interest groups to achieve systematic consensus as to the prioritisation of methodological uncertainties in this area.^19^ This protocol presents the rationale and process for the planned Delphi study.

## Materials and Methods

### Aims

This study aims to systematically identify the 10 most important methodological uncertainties, in the design, conduct and analysis of trials that aim to improve the care of people living with MLTCs, which should be addressed within the next 5 years.

### Study design

We will conduct a four-stage modified Delphi study^20^ including methodologists, trialists, MLTC researchers, people with lived experience of MLTCs and MLTC carers to identify and prioritise methodological research uncertainties in the design, conduct and analysis of MLTCs trials. We are using a modified Delphi approach that allows participants to join the study in different rounds to encourage inclusivity and capture a broad range of perspectives. This flexible design is particularly valuable for incorporating diverse expertise and experiences particularly from participants who may face barriers to joining all rounds of the Delphi.^21^ Alongside this we will give those living with MLTCs and their carers an additional opportunity to input into these priorities through externally facilitated meetings (one online and one in person meeting).

This modified Delphi consensus study will be conducted and reported according to the Conducting and REporting of DElphi Studies (CREDES) recommendations and the RAND Methodological Guidance.^20,22^

### Research steering group

A research steering group has been appointed to oversee the progress of the study, including developing and agreeing on the protocol, reviewing results after each round, and finalising the list of research uncertainties. This group will include the research leadership team (Laura Gray, Kamlesh Khunti, Natalie Darko and Sally Singh) and a number of independent members (Sharon A Simpson, Susan Smith, Rod Taylor, Miles Witham and Hannah Young), as well as our PPI Advisory Group ((PAG) see below).

### Eligibility and recruitment

Adults aged 18 years and over with an interest in trials and/or MLTCs, including those with lived experience of MLTCs and their carers, will be eligible to take part in the study. There will be no restriction on country of residence. We have identified relevant groups of interest which are listed in Table 1, along with the recruitment strategy for each. Alongside these we will use our personal and institutional social media accounts to advertise the opportunity to take part. Our Research Steering Group will also identify eligible individuals who will be sent a personalised letter of invitation via email.

**Table 1.** Interest group identification and recruitment.

Groups of interest	Definition	Recruitment strategy
Trialists & Methodologists	Researchers of all career stages working at academic or private organisations with an interest in the methodological aspects of trials.	• Medical Research Council (MRC)- National Institute for Health and Care Research (NIHR) Trials Methodology Research Partnership https://www.methodologyhubs.mrc.ac.uk/about/tmrp/
		• Health Research Board (HRB)-Trials Methodology Research Network. hrb-tmrn@universityofgalway.ie
		• Accredited Clinical Trial Unit (CTU) network: UKCRC Registered CTUs https://ukcrc-ctu.org.uk/contact-us/
		• AllStat info@allstats.co.uk
		• NIHR Research Support Service https://www.nihr.ac.uk/support-and-services/research-support-service
		• Population Health Sciences Institute, Ridley, Newcastle University statisticsgroup@nihr.ac.uk
Multiple Long-Term Condition (MLTC) researchers	Clinical or non-clinical researchers of all career stages working at academic or private organisations involved in health and social care, or direct patient care and with an interest in MLTCs.	• International Research Community in Multimorbidity jose.almirall@usherbrooke.ca
		• NIHR relevant infrastructure (Applied Research Collaborative (ARC)/Biomedical Research Centres (BRC) network) arc-em@le.ac.uk
		• MLTCs Cross NIHR Collaboration mltc-cnc@nihr.ac.uk
		• NIHR Global Centre in MLTCs sc833@leicester.ac.uk
		• Society for Academic Primary Care office@sapc.ac.uk
		• British Geriatrics Society enquiries@bgs.org.uk
		• Authors of recent relevant MLTC publications and those identified by our systematic review.^15^ (20 Authors)
		• Authors of ongoing MLTC trials from clinical trial registries. (82 Authors)
Funding bodies, policy makers, regulating bodies and commissioners	Funders of MLTC trials and NICE.	• National Institute for Health and Care Research (NIHR) academy@nihr.ac.uk
		• Medical Research Council (MRC) corporate@mrc.ukri.org
		• National Institute for Health and Care Excellence (NICE) nice@nice.org.uk
		• Integrated care boards
People living with MLTCs and their carers	Those with lived experience of MLTCs and their carers	• PPI (Patient Public Involvement) groups associated with the NIHR BRC Leicester and NIHR ARC East Midlands
		• People in Research (NIHR) https://www.peopleinresearch.org/
		• Be Part of Research (NIHR) https://bepartofresearch.nihr.ac.uk/
		• Existing PPI groups
		• Communities associated with the study’s PAG members.
		• Convenors of MLTC PPI groups will be asked forward our invite to their members.

Mail outs, advertisements and personal invitations customised for specific groups of interest will include a link to get additional information and join the study. The link will include information about the purpose of the study and how participant data will be used. Those wanting to take part will then confirm their consent through a tick box form and proceed to the questionnaire. Participants will be given the option to provide their email address to be contacted about the next rounds and to receive a summary of the study results. Any email addresses provided will be stored separately from the questionnaire responses. This process will be repeated for each round.

Participants will then be asked about their characteristics so that the representativeness of the respondents can be summarised. We will collect data on

• Group of interest
  o For those with MLTCs, we will collect whether their conditions are physical, mental or infections and how many conditions they have.
• Country of residence
• Age group
• Gender
• Ethnicity
• Profession (if relevant)
• Sector (if relevant)

### Sample size

There is little guidance on the sample size requirements for Delphi studies, with sample size depending on group dynamics for arriving at a consensus among experts rather than statistical power.^22^ The average size reported is between 15-20 participants. Other studies have shown that a sample size of 23 is sufficient if the participants included have similar training and general understanding in the field of interest.^23,24^ A recent Delphi study on the definition and measurement of MLTCs recruited 150 professional participants.^25^ We will aim for no fewer than 30 participants in Round 1 and 2, but will have no maximum. Round 3 (the lived-experience meeting) and Round 4 (the online-consensus meeting), will each be held with a maximum 30 participants. We will purposefully try to capture a range of expertise, by utilising specific professional bodies for sending out invites.

### Consensus process

**Figure 1.**
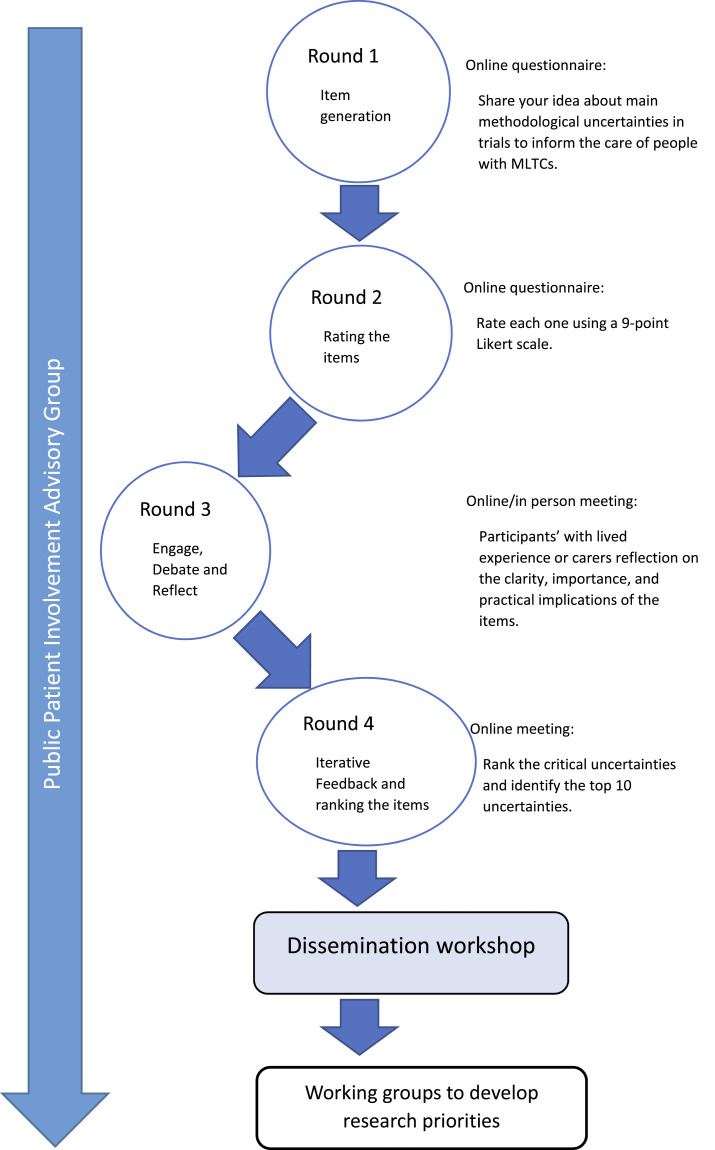
Delphi study flow diagram.

The consensus process will have four rounds: two administered by online questionnaires, one virtual/in person lived experience meeting and one virtual consensus meeting (Figure 1).

The online questionnaires will be developed with our Patient and Public Advisory Group (PAG) and piloted to ensure the instructions given are clear and the process is acceptable. Amendments will be made after the pilot. Piloting will be undertaken with local researchers who are not involved in this project. Pilot entries will be disregarded. We will recruit participants from the sources listed in Table 1. All participants (public and professional) will be encouraged to forward our invite to their networks. This snowballing technique was successfully used in the previous consensus study.^25^

#### Round 1

An electronic invitation letter will be shared with potential participants via social media, relevant organisations, personal emails, and public and professional networks. The letter will include links to the Participant Information Sheet, the study’s privacy notice, and a one-page summary of current approaches to the design and analysis of MLTC trials derived from our recently completed systematic review.^15^ The invitation letter will also include a link directing readers to the electronic informed consent page. After agreeing to participate and providing informed consent, the survey questions will become available to participants. The survey will begin with a small number of questions capturing participants’ relevant characteristics. Participants will then be asked three open-ended questions about what they consider to be the main methodological uncertainties when designing, conducting and analysing trials to inform the care of people with MLTCs. Based on feedback from our PAG, detailed explanations of the questions in lay language will also be provided. Free text boxes with no maximum length will be used for these responses so that participants can list as many responses as they wish. Respondents will be encouraged to share any thoughts they consider relevant, without limiting their answers. Round 1 will be open for 6 weeks.

Once completed, the text responses will be assessed to identify methodological themes that are specifically related to MLTC trials. All issues identified by participants in Round 1 that are relevant to MLTC trials, whether they affect all MLTC trials or only subsets of trials (for example those assessing models of care), will be carried forward to Round 2. Overall themes with specific methodological uncertainties within each theme will be summarised. The study investigators and steering group will review the results.

#### Round 2

After completing the analysis of Round 1 data, the invitation process will be repeated. New participants will be invited alongside participants from Round 1 who have expressed an interest in taking part in Round 2.

In this round, the methodological uncertainties identified in Round 1 will be presented to participants by theme. It will be clarified that the term “priority” refers to methodological uncertainties that have the greatest impact on the validity or feasibility of MLTC trials and that should be addressed in future methodological research. Themes and uncertainties will be presented in a random order. Participants will be asked to rate each uncertainty using a 9-point Likert scale. A score of 1-3 will indicate an uncertainty is “Not important”, 4-6 will indicate “Important, but not critical”, and 7-9 will indicate an uncertainty is “Critical”. Participants will also be able to select ‘unable to score’ if they felt unable to offer an opinion on a particular uncertainty; this will be assigned a score of 0. Participants will also be able to add any uncertainties they feel were not captured in Round 1. Round 2 will be open for 6 weeks.

The responses from Round 2 will be analysed using summary statistics to determine consensus status for each uncertainty. Median and interquartile range (IQR: from the 25th to the 75th percentile) of each uncertainty will be used to set the consensus status, see Table 2. Depending on the number of participants in each main group (professional or lived-experience participants, gender, age group, and country of residence), exploratory subgroup analyses may be considered.

**Table 2.** Consensus criteria.

Criteria	Median	IQR^1^	% Agreement^2^	Interpretation
Strong Agreement	7–9	≤ 2	≥ 70%	Clear consensus on importance/relevance
Moderate Agreement	7–9	> 2	50–69%	No consensus, more discussion needed on importance of the item.
Uncertain/Neutral	4–6	Any	Any	No strong opinion; item may need revision
Strong Disagreement	1–3	≤ 2	≥ 70%	Clear consensus that the item is not important
Divergent Opinions	1–3	> 2	< 70%	No consensus, more discussion is needed on the (un)importance of the item.

^1^The IQR represents the middle 50% of participants’ scores (from the 25th to the 75th percentile).

^2^The agreement is the percentage of participants whose scores fall within the defined range for median.

#### Round 3

PPI contributors have highlighted potential challenges that individuals with lived experience of MLTCs and their carers might face when completing online questionnaires. In response, round 3 has been integrated into the study to provide an additional opportunity for PPI participants to contribute and ensure their perspectives are meaningfully represented. Thirty people living with MLTCs or their carers who declare their interest in participating will be invited to Round 3. Participants will be selected to aim for balanced representation across gender and ethnic groups.

Round 3 will consist of a single meeting. Participants will be invited to attend a facilitated meeting, either in person or virtually. These sessions will serve to present the study findings, particularly items falling into the moderate agreement, uncertain/neutral or divergent opinion categories in Round 2, and to explore their relevance and resonance with those directly affected by MLTCs.

Each session will begin with an accessible overview of the consensus process, followed by a presentation of the uncertainties without consensus using plain language and visual aids. Facilitated small-group discussions will then allow participants to reflect on the clarity, importance, and practical implications of the items from their lived experience perspective. They will also be able to rate items using live voting. Insights gathered during these sessions will be incorporated with those from Round 2 to ensure the identified research uncertainties accurately reflects the views and values of people living with MLTCs and their carers. The findings of Round 3, including votes and qualitative feedback for each item will be summarised and presented at the Round 4 meeting to inform voting in Round 4.

#### Round 4

Round 4 will consist of an online consensus meeting to rank the critical uncertainties and identify the 10 most important uncertainties which should be addressed within the next 5 years. We will ask for expressions of interest to attend from participants who provided their email addresses in earlier rounds, with additional recruitment as needed to ensure balanced representation across interest groups. From the pool of interested participants, a purposive sample will be selected to include all interest groups and individuals with lived experience and carers. Selection will aim to maximise diversity across interest groups and participant characteristics. Up to 30 participants will be invited to take part in the meeting. If more volunteers with the same characteristics are available, those who participated in previous rounds will be given priority.

The online consensus meeting will be run by an experienced external facilitator to a pre agreed agenda. At the beginning of the meeting, the study objectives will be reviewed, and definitions of the terms “methodological uncertainty” and “priority” will be provided to ensure consistency. Each critical uncertainty will be described in an accessible way and the data from the previous rounds summarised. Online live voting will be used during the meeting to rank the uncertainties. Consensus will be defined as ≥70% of panellists assigning a rating of ≥7 or ≥70% of panellists assigning a rating of ≤3, provided that the item has an IQR ≤ 2. Consensus and non-consensus items can relate to either importance or unimportance. Items with a median between 4 and 6 will be reported as: “No strong opinion regarding how important this item is.” (Table 2). Items that do not meet this criterion will be discussed and revoted up to three times. Items that do not reach consensus will be reported separately with a brief of discussion of why participants thought differently about that item.

For consensus items, the median rank will be used to produce an initial prioritised list. When two or more items have the same median rank, the uncertainty associated with the median rank will be considered. First, items with a smaller IQR, and then items with a higher agreement rate (percentage of participants within ±1 position of the median) will be assigned the higher rank. Analyses will initially be conducted across all participants, regardless of their interest group or personal characteristics.

### Patient and public involvement

This modified-Delphi study is part of a larger programme of work which includes a systematic review and dissemination workshop. We have established a PAG, comprised of 8 members including people with MLTCs and their carers. The PAG will meet at least 3 times during the research programme, approximately every 4 months, with a mix of online and face-to face meetings (with a hybrid option). The PAG have advised on recruitment for the consensus study and research plans and will input into the findings to ensure our work remains relevant to those living with MLTCs.

### Dissemination plan

We will hold an online dissemination workshop to share the findings of the study. We have chosen to hold this as an online meeting to maximise the attendance from a diverse audience. At the workshop the results of the systematic review^15^ and consensus study will be presented. As suggested by the PAG, attendees will be able to join breakout rooms for the particular priority questions they have an interest in. The breakout room discussions will be facilitated to enable people to network with others who have similar interests and start to brainstorm how each question could be addressed. There will be opportunities for attendees to attend breakout rooms for up to three questions. Members of the PAG and the research team will facilitate the breakout rooms and there will be time to feedback the main outcomes of these discussions. To record the event, live scribing will be used as a visual record of the key outcomes. The dissemination workshop will serve two purposes: first, to present the study findings to interested groups; and second, to encourage networking among these groups, including researchers and PPI contributors, to address the identified uncertainties in their future methodological research or to consider them in the next trials to which they contribute. After the event we will ask for permission to share contact details of attendees so that collaborations can continue. We will also organise follow up meetings where requested.

In addition to the workshop and follow-up meetings, the authors, who are members of the NIHR Multiple Long-Term Conditions Cross-NIHR Collaboration, will facilitate the dissemination of the results. The study findings will be published in a peer-reviewed journal and presented at relevant conferences.

### Data management plans

All data will be collected and managed via REDCap electronic data capture tool hosted at University of Leicester^26,27^ and stored securely on password-protected servers owned by the University of Leicester.

Personal data will be separated from the main datasets and will only be accessible by the research team. Once the study is completed, the personal data will be deleted permanently. Participants’ contact information will be deleted unless they provide consent to retain it for further follow-up, if necessary.

Participants will be assured that their information will be kept confidential. All feedback provided in each round will be presented as aggregated distributions, ensuring that no individual responses are disclosed. Participants will also be informed that they can withdraw from the study at any time but that, depending on the timing of their withdrawal, we may not be able to remove their data.

### Strategies to avoid bias during the validation process

Responses will remain anonymous to other participants throughout the consensus process. In all four rounds, no weighting or preferential treatment will be applied based on interest group or other participant characteristics. Analyses will be conducted and finalised in each round without access to individual responders’ personal information.

### Potential risk and risk management

The most important risk for this study is low recruitment or a significant imbalance between the number of professionals and public participants. To address this, we will use purposive and snowball sampling, send personalised invitations, and clearly communicate the study’s objectives and time commitments. The study team and steering group have extensive networks and collaborations in this area, and it is hoped that through this recruitment will be maximised. If recruitment is low, we will extend the study rounds until we meet our minimum sample size.

Another challenge is the potential for high participant dropout between rounds. While our consensus approach allows recruitment of new participants between rounds, a large number of dropouts and newcomers could hinder reaching consensus. To mitigate dropout, we will make participation easy and user-friendly, provide support for participants who request explanations or clarifications, and assure confidentiality of their contact information on the invitation page. Additionally, we will offer acknowledgments in the resulting publication.

In Rounds 1, 2, and 3, participants will be asked whether they are interested in participating in the next round(s). Therefore, we will have a group of individuals whom we can approach directly, in addition to the general distribution of invitations. For social networks, specific organisations, professional and PPI groups to which we distribute the link, we will send reminders one week before the deadline.

Many MLTC-focused clinical trials have been conducted in the UK, and the sampling approach in this study will most likely involve UK-based researchers and PPI participants. Therefore, we expect that the majority of methodological uncertainties identified will reflect the UK context. To increase the likelihood of recruiting international participants, we will contact the International Research Community in Multimorbidity, authors of relevant MLTC publications, individuals identified through our systematic review,^15^ and investigators of ongoing MLTC trials from clinical trial registries, including lead authors of published MLTC trials. In addition, the authors will share the invitation link through their networks at the NIHR Multiple Long-Term Conditions Cross-NIHR Collaboration and the NIHR-funded Global Centre for MLTCs based in India and Nepal.

### Ethical considerations

All participants must provide informed consent prior to participating. A Participant Information Sheet will be provided in the invitation email. Participants are required to open and read through this document in full before being allowed to access the consent form. The project has received ethical approval from the University of Leicester.

## Discussion

This protocol describes the design of a modified-Delphi study to obtain consensus from professionals and people with lived experience on the top methodological uncertainties in the design, conduct and analysis of trials to inform the care of people with MLTCs.

Currently, there is limited research available to guide study design and conduct in this challenging and evolving area. Trial advancement was emphasised in the National Institute of Health and Care Research (NIHR) strategic framework for MLTCs, with methodologies being identified as one of the strategic priority areas. The NIHR have specifically noted the need for outcome frameworks and ensuring representation within MLTC trials.^28^

We will use a modified Delphi approach to build consensus among professionals and individuals with lived experience regarding methodological uncertainties across the landscape of trial design, conduct, and analysis in MLTC trials. Some uncertainties may be broadly applicable, while others may be specific to particular trial types. Our approach is intentionally inclusive, allowing participants with experience across different MLTC trial types to contribute, ensuring that the final prioritised list reflects the full spectrum of methodological challenges and identifies key areas for improvement.

We have selected a modified-Delphi design as a cost-effective way to gather the opinions from a large, representative and diverse group of both professional and public contributors.^20,23^ In a similar study, Ho and colleagues (2021) conducted a three-round online Delphi study involving 150 professionals and 25 individuals with LTCs to develop consensus on the definition and measurement of MLTC.^25^ Although we have set a low minimum sample size, we will have no maximum for the online questionnaires and will focus on both diversity as well as the overall number of responses.

To capture diverse perspectives from people who live with MLTCs, and interested professionals, we plan not to restrict participation in each round to those who took part in previous rounds. Although this approach does not completely align with the iterative feedback process among a relatively stable panel in conventional Delphi studies, we decided to allow inclusive input in Round 2 for the list of uncertainties identified in Round 1, without excluding contributions from participants who did not take part in Round 1. Round 3 provides an opportunity to collect additional input, and in this round, contributions from any interested participants are also considered valuable. In Round 4, the focus shifts to achieving consensus through iterative vote–feedback–vote cycles, enhancing the stability of responses among a core group of participants. This approach was recently applied by Williams et al. (2023) in prioritising methodological considerations for using routine data in clinical trial studies.^21^

A key strength of this study is our commitment to creating an inclusive environment of a diverse group of participants who all feel comfortable contributing. We will offer one-on-one meetings with our public contributors in advance to ensure they feel prepared and have the opportunity to ask any questions. This will allow them to fully engage in the process.

To further reduce power imbalances and encourage participants to share their views, our lived experience meetings will be co-facilitated by Nifty Fox Creative and a member of our PAG. Nifty Fox Creative is an agency that specialises in communicating complex ideas in creative and accessible ways, and we have successfully collaborated with them before, including on a co-produced animation explaining the importance of PPI in methodological research.^29^

## Conclusion

This protocol outlines the design of a modified-Delphi approach to achieve consensus among professionals and individuals with lived experience on methodological uncertainties in trials for people with MLTCs. In response to the NIHR’s strategic emphasis on advancing MLTC research, particularly in methodology and inclusivity, our study aims to identify key areas for improvement in trial design, conduct and analysis. By adopting a flexible and inclusive Delphi method, supported by creative facilitation and direct engagement with public contributors, we seek to ensure that diverse perspectives are meaningfully incorporated. This approach is intended to support the development of more patient-centred, representative, and methodologically sound trials in the MLTC field.

## References

[1] MacMahon S Calverley P Chaturvedi N , et al.. Multimorbidity: a priority for global health research. The Academy of Medical Sciences, 2018, 127.

[2] Imison C . Multiple long-term conditions (multimorbidity): making sense of the evidence. National Institute for Health Research 2021. https://evidence.nihr.ac.uk/collection/making-sense-of-the-evidence-multiple-long-term-conditions-multimorbidity/

[3] Khunti K Sathanapally H Mountain P . Multiple long term conditions, multimorbidity, and co-morbidities: we should reconsider the terminology we use. BMJ: British Medical Journal (Online) 2023; 383: p2327. 10.1136/bmj.p2327 37832952

[4] Mercer S Furler J Moffat K , et al. Multimorbidity: technical series on safer primary care. World Health Organization, 2016.

[5] Boast J . Making more of multimorbidity: an emerging priority. Lancet 2018; 391: 1637. 29726322 10.1016/S0140-6736(18)30941-3

[6] Valabhji J Barron E Pratt A , et al. Prevalence of multiple long-term conditions (multimorbidity) in England: a whole population study of over 60 million people. Journal of the Royal Society of Medicine 2024; 117: 104–117. 10.1177/01410768231206033 37905525 PMC11046366

[7] Hayanga B Stafford M Saunders CL , et al.. Ethnic inequalities in age‐related patterns of multiple long‐term conditions in England: Analysis of primary care and nationally representative survey data. Sociology of Health & Illness 2024; 46: 582–607. 10.1111/1467-9566.13724 37879907

[8] Chudasama YV Khunti K Gillies CL , et al. Healthy lifestyle and life expectancy in people with multimorbidity in the UK Biobank: a longitudinal cohort study. PLoS medicine 2020; 17: e1003332. 10.1371/journal.pmed.1003332 32960883 PMC7508366

[9] Steell L Krauth SJ Ahmed S , et al. Multimorbidity clusters and their associations with health-related quality of life in two UK cohorts. BMC medicine 2025; 23: 1. 10.1186/s12916-024-03811-3 39773733 PMC11708164

[10] Islam N Shabnam S Khan N , et al. Combinations of multiple long term conditions and risk of hospital admission or death during winter 2021-22 in England: population based cohort study. BMJ medicine 2024; 3: e001016. 10.1136/bmjmed-2024-001016 39574426 PMC11580288

[11] Krauth SJ Steell L Ahmed S , et al. Association of latent class analysis-derived multimorbidity clusters with adverse health outcomes in patients with multiple long-term conditions: comparative results across three UK cohorts. Eclinicalmedicine 2024; 74: 102703. 10.1016/j.eclinm.2024.102703 39045545 PMC11261399

[12] Smith SM Wallace E Clyne B , et al. Interventions for improving outcomes in patients with multimorbidity in primary care and community setting: a systematic review. Systematic Reviews 2021; 10: 1–23. 10.1186/s13643-021-01817-z 34666828 PMC8527775

[13] Weiss CO Varadhan R Puhan MA , et al. Multimorbidity and evidence generation. Journal of general internal medicine 2014; 29: 653–660. 10.1007/s11606-013-2660-5 24442333 PMC3965759

[14] Stoll CR Izadi S Fowler S , et al. Multimorbidity in randomized controlled trials of behavioral interventions: A systematic review. Health Psychology 2019; 38: 831–839. 10.1037/hea0000726 31045382 PMC6983953

[15] Zhang L Fisher E Bradbury N , et al. Randomised controlled trials for improving health outcomes for people living with multiple long-term conditions: Protocol for a systematic review of methodological approaches, risk of bias and reporting quality. Plos one 2025; 20: e0325742. 10.1371/journal.pone.0325742 40587473 PMC12208475

[16] UK NGC . Multimorbidity: assessment, prioritisation and management of care for people with commonly occurring multimorbidity, 2016. 27683922

[17] Smith SM Bayliss EA Mercer SW , et al. How to design and evaluate interventions to improve outcomes for patients with multimorbidity. Journal of comorbidity 2013; 3: 10–17. 10.15256/joc.2013.3.21 29090141 PMC5636021

[18] Stokes J Bower P Smith SM , et al. A primary care research agenda for multiple long-term conditions: a Delphi study. British Journal of General Practice 2023; 74: e258–e263. 10.3399/BJGP.2023.0163 PMC10947355 38164536

[19] Humphrey-Murto S De Wit M . The Delphi method—more research please. Journal of clinical epidemiology 2019; 106: 136–139. 10.1016/j.jclinepi.2018.10.011 30352274

[20] Khodyakov D Grant S Kroger J , et al.. RAND methodological guidance for conducting and critically appraising Delphi panels. Rand, 2023.

[21] Williams AD Davies G Farrin AJ , et al. A DELPHI study priority setting the remaining challenges for the use of routinely collected data in trials: COMORANT-UK. Trials 2023; 24: 243. 10.1186/s13063-023-07251-x 36997954 PMC10064573

[22] Jünger S Payne SA Brine J , et al. Guidance on Conducting and REporting DElphi Studies (CREDES) in palliative care: Recommendations based on a methodological systematic review. Palliative medicine 2017; 31: 684–706. 10.1177/0269216317690685 28190381

[23] Okoli C Pawlowski SD . The Delphi method as a research tool: an example, design considerations and applications. Information & management 2004; 42: 15–29. 10.1016/j.im.2003.11.002

[24] Akins RB Tolson H Cole BR . Stability of response characteristics of a Delphi panel: application of bootstrap data expansion. BMC medical research methodology 2005; 5: 1–12. 10.1186/1471-2288-5-37 16321161 PMC1318466

[25] Ho IS Azcoaga-Lorenzo A Akbari A , et al. Measuring multimorbidity in research: Delphi consensus study. BMJ medicine 2022; 1: e000247. 10.1136/bmjmed-2022-000247 36936594 PMC9978673

[26] Harris PA Taylor R Thielke R , et al. Research electronic data capture (REDCap)—a metadata-driven methodology and workflow process for providing translational research informatics support. Journal of biomedical informatics 2009; 42: 377–381. 10.1016/j.jbi.2008.08.010 18929686 PMC2700030

[27] Harris PA Taylor R Minor BL , et al. The REDCap consortium: building an international community of software platform partners. Journal of biomedical informatics 2019; 95: 103208. 10.1016/j.jbi.2019.103208 31078660 PMC7254481

[28] (NIHR) NIfHaCR . NIHR Strategic Framework for Multiple Long-Term Conditions. NIHR; 2022. https://www.nihr.ac.uk/nihr-strategic-framework-multiple-long-term-conditions 2024, accessed 16 June 2025.

[29] NIHRtv . What is statistical methodology research and why is PPIE input important? (2023). https://www.youtube.com/watch?v=4rzEHbA4p48

